# The H19 Non-Coding RNA Is Essential for Human Tumor Growth

**DOI:** 10.1371/journal.pone.0000845

**Published:** 2007-09-05

**Authors:** Imad J. Matouk, Nathan DeGroot, Shaul Mezan, Suhail Ayesh, Rasha Abu-lail, Abraham Hochberg, Eithan Galun

**Affiliations:** 1 Department of Biological Chemistry, Institute of Life Sciences, Hebrew University, Jerusalem, Israel; 2 Goldyne Savad Institute of Gene Therapy, Hadassah Hebrew University Hospital, Jerusalem, Israel; Universität Heidelberg, Germany

## Abstract

**Background:**

Mutations and epigenetic aberrant signaling of growth factors pathways contribute to carcinogenesis. Recent studies reveal that non-coding RNAs are controllers of gene expression. H19 is an imprinted gene that demonstrates maternal monoallelic expression without a protein product; although its expression is shut off in most tissues postnatally, it is re-activated during adult tissue regeneration and tumorigenesis. Moreover, H19 is highly expressed in liver metastasis derived from a range of carcinomas. The objective of this study is to explore the role of H19 in carcinogenesis, and to determine its identification as an anti-tumor target.

**Methodology/ Principle Findings:**

By controlling oxygen pressure during tumor cell growth and H19 expression levels, we investigated the role of H19 expression in vitro and in vivo in hepatocellular (HCC) and bladder carcinoma. Hypoxia upregulates the level of H19 RNA. Ablations of tumorigenicity of HCC and bladder carcinomas in vivo are seen by H19 knockdown which also significantly abrogates anchorage-independent growth after hypoxia recovery, while ectopic H19 expression enhances tumorigenic potential of carcinoma cells in vivo. Knocking-down H19 message in hypoxic stress severely diminishes p57^kip2^ induction. We identified a number of potential downstream targets of H19 RNA, including angiogenin and FGF18.

**Conclusions:**

H19 RNA harbors pro-tumorigenic properties, thus the H19 gene behaves as an oncogene and may serve as a potential new target for anti-tumor therapy.

## Introduction

The phenotypic and gene expression similarities of tumor cells to cells at different developmental stages are becoming apparent. One specific example is the liver, in which hepatocytes, upon malignant transformation, express developmentally regulated genes such as α-fetoprotein. Hepatocellular carcinoma (HCC) is the third leading cause of worldwide cancer deaths, most of those (>79%) occurring in Asia. The etiology of the overwhelming majority of HCC cases is associated with a chronic inflammatory process, suppression of apoptosis with an enhanced ongoing cell cycle activity. Although considerable effort has focused on unraveling the molecular pathogenesis of HCC during the last few years, constructive knowledge remains mostly unknown [1]. The application of array-based, high-throughput genomic technologies to measure global gene expression, chromosomal alterations, and mutations have started to provide comprehensive information on the molecular pathogenesis of human HCC [2]. A tumor progression model for human HCC based on bioinformatic analysis of genomic data was able to identify three subgroups of patients with different degrees of tumor progression [3]. It is apparent that hepatocarcinogenesis is dependent on numerous genetic alterations [4]. Some of these changes could be linked to specific etiological factors, including integration of the hepatitis B virus (HBV) DNA, leading to chromosomal instability, insertional instability or alteration of cellular gene expression, in particular the HBV X transactivating gene. Aflatoxin B1-exposed patients induces R249S mutation by an adduction mechanism in the tumor suppressor protein p53 [5]. Vinyl chloride exposure induces mutations in K-Ras, hepatocyte nuclear factor 1α (HNF1 α) mutations associated with hepatocellular adenomas and adenomatosis polyposis coli (APC) germline mutations predisposing to hepatoblastomas. In addition, there are numerous genetic alterations which are etiologically nonspecific, including recurrent gains and losses of chromosomes, alteration of TP53 gene, activation of WNT/β-catenin pathway through CTNNB1/ β-catenin and AXIN (axis inhibition protein) mutations, inactivation of retinoblastoma and IGF2R (insulin-like growth factor 2 receptor) pathways through inactivation of RB1 (retinoblastoma 1), P16 and IGF2R. Furthermore, specific signaling pathways are also tightly associated with subsets of HCC natural history. The MET tyrosine-kinase receptor is a sensor of adverse microenvironmental conditions (such as hypoxia) that drives cell invasion and metastasis through the transcriptional activation of a set of genes [6]. Applying global gene expression profiling of wild type and Met-deficient primary mouse hepatocytes facilitated the identification of the Met-dependent gene expression signature within a subset of HCC with poor prognosis and aggressive phenotype [7]. We, as others, have also applied the global gene expression profiling to unfold the specific signaling associated with inflammation-induced HCC in the MDR2 knockout mice [8]. In this specific model of inflammation-induced tumors, we provided evidence that NFκB has a role in HCC progression [9]. Moreover, we have previously reported on the role of additional inflammatory signaling and microenvironment in tumor progression [10]. The importance of liver inflammation was also reported by others, suggesting alternative mechanisms for HCC development dependent on liver residing inflammatory cells and innate immune signaling [11]. In one specific case of inflammation-associated tumors, our group reported that in HBV infection-associated HCC, there was an increased level of the imprinted , non-coding H19 RNA , suggesting its use as a tumor marker [12].

During the past few years, a wealth of information revealed the importance of different types of non-coding RNAs in controlling gene expression. X–inactivation is controlled by the non–coding RNA transcript Xist through an epigenetic mechanism [13]. The small non–coding microRNAs can be perceived as tightly linked to carcinogenesis [14]. The H19 gene transcribes also to a non–coding RNA [15]. H19 is an imprinted oncofetal gene that demonstrates maternal monoallelic expression in fetal tissues, and does not code for a protein. It is abundantly expressed in embryogenesis, but shut off in most tissues after birth. The imprinted cluster on the human chromosome 11p15.5 has been implicated in a variety of disorders and cancer predisposition for both pediatric and adult tumors. This observation initially places the H19 gene as a candidate gene that fulfills a role in tumorigenesis. H19 overexpression promotes tumorigenic properties of breast cancer cells in vivo [16]; its expression is necessary for cell entry into S-phase after serum deprivation by E2F1 binding to its promoter [17], and is negatively regulated by p53 [18]. Recently, it was shown that H19 repression was tightly associated with de-differentiation of cells compared to undifferentiated parental cells [19]. Furthermore, in the first report on the importance of the Twist gene product in tumor metastasis, the investigators showed that H19 is the highest differentially expressed gene in metastatic tumor cell lines investigated [20]. Certain known carcinogens upregulate the level of the H19 RNA. In a study aimed to identify changes in gene expression patterns in the airway epithelium of disease-free smokers compared with a matched group of nonsmokers, a dramatic elevation of H19 RNA levels was detected in the airway epithelium of smokers without affecting loss of imprinting [21]. We have shown that N-Butyl-N-(4-hydroxybutyl) nitrosamine (BBN) (a known carcinogen of the bladder) added to the drinking water also induces the expression of H19 gene in a rat model of bladder cancer in early stages [22], [23]. Moreover, diethylnitrosamine (a known carcinogen of the liver) induces the expression of H19 RNA in a mouse model of HCC [24]. More striking is the predictive value of H19 RNA for tumor recurrence, and its prognostic significance [25]. H19 is expressed in both epithelial and stromal components of human invasive breast adenocarcinoma; in contrast , it was reported that of all tumors of breast adenocarcinoma displaying a good prognosis (grade I), only the stromal component expresses H19 [26].

Two recent reports have linked indirectly H19 to HCC development: **1.** c-Myc induced the expression of the H19 RNA. c-Myc binds to the E-boxes near the imprinting control region to facilitate histone acetylation and transcriptional initiation of the H19 gene . c-Myc also down-regulates the expression of IGF2, the reciprocally imprinted gene at the H19/IGF2 locus [27]. c-Myc upregulation is an important factor in HCC development [28] as well as in many other tumors. **2.** The H19 is reported to be a target gene for the hepatocyte growth factor (HGF), further signifying the potential role of H19 RNA in HCC development [29]. Interestingly, H19 RNA is upregulated in HBV-associated HCC [30]. Furthermore, a biallelic expression of H19 gene was found in human HCC patients [31] and in liver neoplasms of albumin SV40 T-antigen-transgenic rats [32].

In the current study, we highlight a critical role of H19 RNA in tumor development. Cognizant of the role of hypoxia in enhancing the signaling through the HGF/c-Met pathway, we investigated as an initial step the effect of hypoxia on H19 expression in HCC. Moreover, our previous results point to a growth advantage role of H19 RNA in serum stress, and modulation of the expression of genes that are linked to angiogenesis [33]. In this study, we show that hypoxia strongly upregulates H19 RNA level. We have also investigated the expression of additional imprinted genes from the H19 gene cluster and showed that knocking down H19 RNA suppresses p57^kip2^ expression. By applying whole genome expression profiling, we showed that H19 knockdown modulates the expression of genes involved in angiogenesis, survival and tumorigenesis in hypoxic stress. We further analyzed the functional consequences of these data and showed that cells that are devoid of H19 expression in hypoxic stress, fail to form colonies in soft agar after hypoxia recovery as opposed to cells that possess H19. Furthermore, silencing H19 expression attenuates tumor growth in vivo. Altogether, these results reveal that H19 harbors an oncogenic activity in the liver through a mechanism that needs further investigations.

## Materials and Methods

### Cell culture

The human carcinoma cell lines (T24P, HepG2, Hep3B, HuH7 and UMUC3 used in this study were obtained from the American Type Culture Collection (Manassas, VA, U.S.A.). The SNU group of HCC cells was provided by M. Ozturk (Bilkent Univer., Ankara, Turkey). HepG2215 (HepG2-HBV producing cell line) was provided by the group of Acs [34] and the FLC4 cells were provided by the Miyamura [35] group; we have generated the FLC4A10 (FLC4-HBV producing cell line) [36] . The cells were maintained in DMEM-F12 (1:1) medium supplemented with 10% fetal calf serum (inactivated at 55°C for 30 min), 25 mM HEPES (pH 7.4), penicillin (180 units/ml), streptomycin (100 µg/ml) and amphotericin B (0.2 µg/ml). Every 4 days, the cells were trypsinized with 0.05% trypsin-EDTA solution (Beit Haemek, Israel) for 10 min and re-plated again using the same initial densities.

### In situ hybridization and immunohistochemistry staining

H19 and α-fetoprotein expression were assessed by using both radioactive and non-radioactive probes for the expression of the H19 gene and immunohistochemistry, for the α-fetoprotein expression according to Ariel et al. [12].

### siRNAs selection and preparation

Four siRNAs targeting human H19 RNA and two negative control siRNAs (targeting luciferase pGL3, or GFP) (Table S1) were synthesized as ready-to-use duplexes by (Invitrogen U.S.A) and designed with dTdT 3′ overhangs on each strand. We selected most H19 siRNA sequences as reported [37]. All sequences were evaluated for gene specificity using the National Institutes of Health Blast program. The freeze-dried siRNAs were dissolved in RNase free- water and stored as aliquots at −80°C.

### Cell culture conditions and transfection of siRNAs

Transfection of siRNAs was conducted with lipofectamine 2000 (Invitrogen, U.S.A.) in 12 well plates. The day prior to transfection, the cells were trypsinized, counted, and seeded at 60,000 cells/well containing 1 ml DMEM medium without antibiotics so that they were nearly at 50% confluence on the day of transfection. Lipofectamine 2000 (3 µl) was incubated for 15 minutes with 100 µl serum-free OPTI-MEM medium (Invitrogen, U.S.A.) and supplemented with 50 pmoles dsRNA diluted in 100 µl serum-free OPTI-MEM media; the formulation lasted 20 minutes. 195 µl of the mixture was applied to the cells and incubated for another 48 hours without replacement of the medium. For hypoxia- mimicking conditions, freshly prepared CoCl_2_ was added at a final concentration of 100 µM, 24 hours post transfection, and the cells were incubated for an additional 22 hours prior to RNA extraction. For hypoxic conditions, Hep3B and UMUC3 cells were seeded and transfected as described above. Twenty four hours post transfection, cells were either placed into an Aneoropack rectangular jar (Mitsubishi Chemical Company, Japan) and supplemented with BBL GasPak Plus (Becton Dickson, Cock-eysville, MD, U.S.A.) to create hypoxic conditions within an hour, or left under normal oxygen concentration. The progression of the hypoxic environment was monitored by a hypoxic indicator. Incubation lasted for an additional 24 hours before RNA extraction.

### RNA extraction and RT-PCR conditions

Reverse transcription of total RNA was performed as described, except that 1 µg total RNA was used [33]. The PCR reaction for H19 was carried out using Taq polymerase (Takara, Otsu, Japan) for indicated cycles (in the legend of each figure) (94°C for 30 s, 58°C for 30 s, and 72°C for 30 s) preceded by 94°C for 5 min, and a final extension of 5 min at 72°C for Hep3B cells and 29 cycles for UMUC3. PCR for Histone, GAPDH, β-actin and for p57^Kip2^ mRNAs was conducted as described [33].

### Ex-vivo tumorigenic assay

For in vivo tumorigenicity, Hep3B and UMUC3 cells were transfected in vitro by two different siRNA duplexes directed against H19 RNA and an unrelated control siRNA (targeting Luc or GFP), respectively as described above. Forty eight hours post transfection, cells were injected subcutaneously into the dorsal flank region of athymic female nude mice (6–8 weeks of age). An additional control group received equal number of untreated Hep3B cells. Cells were trypsinized, counted, and centrifuged and re-suspended into sterile PBS (1X), so that there were about 5×10^6^ cells/ml. 250 µl of the suspension was injected into the dorsal flank region of athymic nude mice. Fifteen and 30 days post injection, tumors began to develop and their volumes were measured using a caliper.

### Anchorage-independent growth

Hep3B cells were seeded and transfected with GFP siRNA and H19 siRNA as indicated above. Four hours post transfection, the cells were placed under hypoxic condition for 24 hours. Cells were washed by PBS, trypsinized, and counted. 2.5×10^3 ^cells were seeded in 6-well plates containing 0.3% top low-melt agarose-0.8% bottom low-melt agarose. Cells were fed every 4 days and colonies were counted microscopically after 4 weeks.

### Cell proliferation analysis

Hep3B cells were seeded and transfected in 12 well plates as described above with GFP siRNA and H19 siRNA. Twenty four hours later, cells were washed twice with PBS, trypsinized and counted. 5×10^3 ^Hep3B cells transfected with GFP or H19 siRNAs were seeded in quadruples in 96 well plates in DMEM media containing 10% FCS, and further incubated for 24 hours before MTS assay was performed. MTS assay was performed according to the procedure provided by the supplier (Promega, USA). The absorbance at 490 nm was recorded using ELISA plate reader.

### Microarray analysis

Hep3B cells were seeded and transfected as described above with H19 and GFP siRNAs. 24 hours later, cells were either exposed to hypoxic stress or continued to grow under normal oxygen cell culture conditions for an additional 24 hours. Total RNAs were isolated 48 hours following siRNAs transfection, and were subjected to reverse transcription, labeling and hybridization to U133A2.0 gene chip arrays (Affymetrix, Santa Clara, CA) containing about 18,400 transcript and variants, including 14,500 well characterized human genes. The siRNAs knockdown experiments were performed non-simultaneously in duplicates. PCR analysis confirmed the upregulation of H19 RNA in hypoxic stress, and H19 knockdown by siRNA (data not shown). Although genes affected could be categorized under different subheadings, two categories will be dealt with here: (1) Genes that fall into the category of hypoxia responsive genes, and are affected by H19 knockdown. (2) Genes that, although not responsive to hypoxia, show modulation of expression by H19 knockdown in hypoxic stress. The significance level was set at a false discovery rate with an ANOVA P-value <0.05. Genes modulated by at least two folds (H19 siRNA versus GFP siRNA in hypoxic stress) are selected. ANOVA analysis identified (87) genes which were significantly differentially expressed falling into these two categories (p<0.05) ((60) up and (27) down).

### Verification of selected genes by semi-quantitative RT-PCR

To verify the different expression levels of mRNA measured by microarray technology, we selected different up- and downregulated genes from the list of differentially expressed genes that are modulated by H19 knockdown. The genes encoding for angiogenin (ANG), insulin-like growth factor binding protein 4 (IGFBP4), v-akt murine thymoma viral oncogene homolog 1 (Akt-1),, fibroblast growth factor 18 (FGF18) and Laminin beta receptor (LBR) were analyzed. Three of them (ANG, FGF18 and Akt-1) falls into the categories described above. The PCR primers sequences are as follows: FGF18 (CCTGCACTTGCCTGTGTTTA For; CAGGGCCGTGTAGTTGTTCT Rev); ANG ( GTGCTGGGTCTGGGTCTGAC For; GGCCTTGATGCTGCGCTTG REV); v-AKT (GTTCTCCGGGTGTGGCCTCAGC For; CCATAGTGAGGTTGCATCTGG TGCC Rev); LBR (GAATTTCCCTCCTCCTTTGC For, CGCGGTCCTGTATTTTCATT Rev ); IGFBP4 (ACCCACGAGGACCTCTACATCATCC For; CAGGCAGAGACAGGACTCAGACTC Rev). All are written from 5′ to 3′ direction. Reverse transcription was performed as indicated above using 1 µg total RNA.

### Reverse-transcription and real time PCR reactions

cDNA was synthesized using 1 µg RNA in a total volume of 20 µl reaction mix using the QuantiTect Reverse Transcription kit (Qiagen), according to the manufacturer's instructions. Relative quantitation of cDNA samples were analyzed using an ABI Prism 7900HT sequence detection system, and the appropriate software (SDS2.2) according to the manufacturer's instructions (Applied Biosystems, 850 Lincoln centre drive, Foster City, CA) and β-actin was used as an internal standard. Two µl of the prepared cDNA was amplified in a mixture of 20 µl containing 0.5 µM primers for the H19 (5′-TGCTGCACTTTACAACCACTG-3′) upstream, (5′ATGGTGTCTTTGATGTTGGGC-3′) downstream, and 0.9 µM of the β-actin primers (5′-CCTGGGACCTGCCTGAACT-3′) forward, (5′-AATGCAGAGCGTCTTCCCTTT-3′) reverse. Whereas 0.1 µM fluorescent probe 6-FAM-TCGGCTCTGGAAGGTTGAAGCTAGAGGA-TAMRA) was used for H19 and 0.25 µM of the beta actin fluorescent probe (6-FAM-TGGTCAGAGAGAGACAC) was used. The PCR conditions consisted of 1 cycle of 2 min at 50°C and 1 cycle of 10 min at 95°C followed by 40 cycles of 95°C for 15 sec, and 60°C for 60 sec. Sensitivity of the QPCR assays: To estimate the sensitivity of the QPCR procedure, a plasmid DNA control which contains part of the H19 cDNA region was used with 10-fold serial dilutions of known quantities from 0.2ng (9×10^7^ copies) to 0.2×10^−7^ ng (≤9 copies of plasmid DNA) for H19 analysis. For β-actin a DNA control was used starting from 0.14ng (7×10^8^ copies) to 0.14×10^−8^ng (≤7 copies). Simultaneous amplifications of standard dilution series were then performed. The number of target copies was determined using the standard curve created in the same run. The QPCR assays were accepted when a positive signal was detected in all positive control dilutions and no signal was detected in the negative sample controls. These experiments were performed in duplicate, at the very least.

### Statistical analyses

Results are presented as mean +/− standard error of the mean. Differences between means were analyzed using the unpaired Student's t-test (two-tailed). None of the animals was excluded from analysis. The probability value p<0.05 was considered statistically significant.

## Results

### H19 expression in HCC

From reports of others and those from our group, it appears that H19 has a role in carcinogenesis. Increased expression of H19 RNA is shown in a large group of tumors (Table S2). HCC is also associated with high H19 expression. In some cases, the high expression is associated with the loss of imprinting (LOI) of the H19 gene. LOI is a term that is conventionally used to describe the change from imprinted monoallelic expression to biallelic expression, and in most cases with an increase of expression. Previous reports have shown the LOI of H19 in many tumor types (Table S3). In specific cases of high H19 expression, we performed an H19 RNA in situ hybridization analysis to depict the level of expression. As could be seen in (Fig. S1), in some cases with H19 increased expression, the H19 message is abundantly expressed in a human HCC tumor, at a much higher level than the traditional HCC marker α-fetoprotein (AFP). As can be seen, AFP is also expressed in the intravascular invasion of HCC, possibly teaching that H19 could serve as a tumor marker. To further assess the expression of H19 in HCC, we determined the level of H19 RNA in a panel of HCC human tumor cell lines. As shown in (Fig. S2A, S2C), H19 RNA is present in most HCC cell lines analyzed, but at different levels. The human HCC cell line, Hep3B, was selected for further analysis based on the observation that H19 is weakly to modestly expressed in this cell line enabling to search for induction conditions as described later. Moreover, H19 RNA is highly increased in Hep3B formed tumors when the cells are subcutaneously injected in the dorsa of nude mice in comparison to its expression in vitro. This also holds true for other panels of cell lines [38], and data not shown.

### H19 RNA is induced in response to hypoxic stress

**Figure 1:**
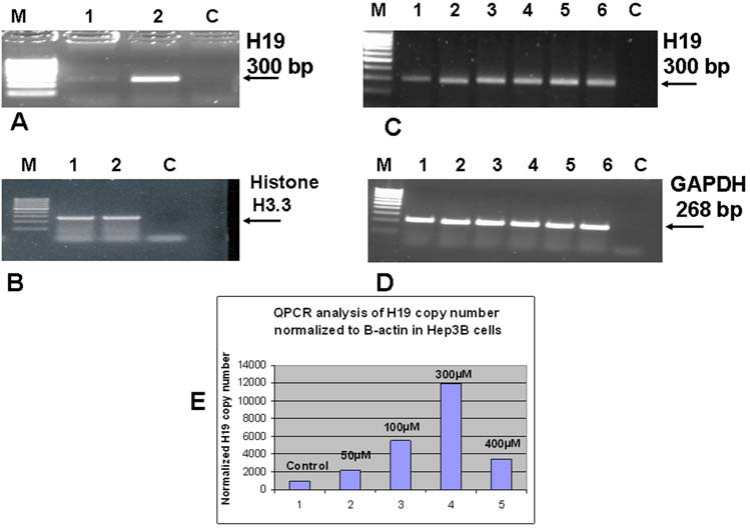
H19 RNA is largely induced in response to hypoxic stress and moderately by hypoxia-mimicking condition triggered by CoCl_2_ in Hep3B. Hep3B cells were cultured under normal culture conditions for 24 hours before hypoxic or CoCl_2_ manipulation. Cells were either placed into an aneoropack rectangular jar to create a hypoxic condition within an hour, or left under normal culture conditions. Incubation lasted for 24 hours before RNA extraction. (A) Shown are RT-PCR products of H19 gene (28 PCR cycles) cultured under normal conditions-lane 1, or hypoxic conditions-lane 2 (lane M indicates the marker, and C is a PCR blank without a target). (B) PCR for Histone H3.3 as a positive control for RT-PCR integrity. Shown also are RT-PCR products of both the H19 gene (32 PCR cycles) (C), the GAPDH gene (D) for untreated Hep3B (lane1) and for 50, 100, 200, 300 and 400 µM CoCl_2_ treated cells (lanes 2, 3, 4, 5 and 6, respectively). Incubation with the indicated concentrations of CoCl_2_ lasted for an additional 22 hours before RNA extraction. QPCR analysis for H19 RNA levels normalized to β-actin in Hep3B treated CoCl_2_ is shown in (E) where the numbers above the bars indicate the concentrations of CoCl_2_ used.

In the HCC cell line, Hep3B, there was an upregulation of H19 RNA in response to hypoxic stress (Fig. 1A). Similar data were obtained in two cell lines of human bladder carcinoma, T24P (data not shown) and UMUC3 (Fig. S5A).

To explore the involvement of the HIFα pathway in the upregulation of H19 RNA during hypoxic stress, we tested whether CoCl_2_, a reagent used to mimic the hypoxic condition, could induce the expression of H19 RNA. Figure 1C shows that H19 gene expression is mildly upregulated in response to the addition of increasing concentrations of CoCl_2_ (50–400 µM). This was also verified using quantitative PCR analyses (Fig. 1E). This moderate upregulation of the H19 RNA by CoCl_2_ relative to its strong upregulation in response to a real hypoxic condition could indicate that the HIF1α pathway might only be partly responsible for this upregulation.

### Knocking down H19 RNA by siRNA in normoxic, hypoxic and hypoxia-like culture conditions

**Figure 2:**
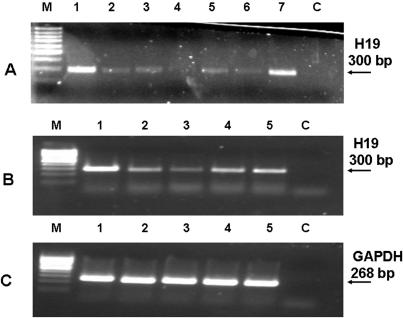
Effect of four different H19 siRNA duplexes on the expression level of H19 in the Hep3B cell line under normal culture condition (A) and hypoxia-mimicking CoCl2 treatment (B). Shown is H19 RNA levels (34 PCR cycles) in (A): Hep3B cells transfected with unrelated siRNA duplex that targets luciferase gene (lane 1) and with 4 different H19 siRNA1 to H19 siRNA4 duplexes (lanes 2–5) and an equimolar mixture of the four siRNAs (lane 6); for controls, we performed transfection also with lipofectamine 2000 without siRNA (Mock) (lane 7) and C is PCR blank without a target. (B): Hep3B cells were transfected under normal medium with siRNA duplex that targets the luciferase gene (lanes 1 and 5) and with 3 different H19 siRNA duplexes (lanes 2–4). Twenty four hours post transfection, media was changed, and 100 µM CoCl_2_ containing media was added except for lane 5 which continued to grow under normal culture media; the incubation lasted for an additional 22 hours. (C): Shown are RT-PCR products of GAPDH gene as a positive control for RT-PCR integrity.

To determine the most potent siRNA to be used for knocking down H19, different H19 siRNAs were transfected into Hep3B cells (Table S1). We examined the ability of these siRNAs to knock-down the endogenous level of H19 RNA under both normal–(normoxic) (Fig. 2A), or hypoxic-like conditions (Fig. 2B). We used four different siRNAs targeting H19 RNA or an equimolar pool of the four siRNAs. Significant decrease to a varying extent of H19 RNA levels was detected by RT-PCR analysis 48 hours post transfection, as compared with non-related PGL3 siRNA duplex targeting luciferase or mock transfected (without siRNA), respectively. The ability of three different H19 siRNAs to suppress the expression of the H19 gene was also tested in hypoxic-like CoCl_2_ simulation (Fig. 2B). While H19 RNA is moderately induced by CoCl_2_ simulation, a very significant reduction is detected using three different siRNA targeting the H19 transcript. Interestingly, the selected H19 siRNA imposed a prolonged silencing effect on the level of H19 message in Hep3B cells (Fig. S3). The silencing effect lasted up to 9 days following transfection with nearly complete suppression shown at least six days after transfection.

**Figure 3:**
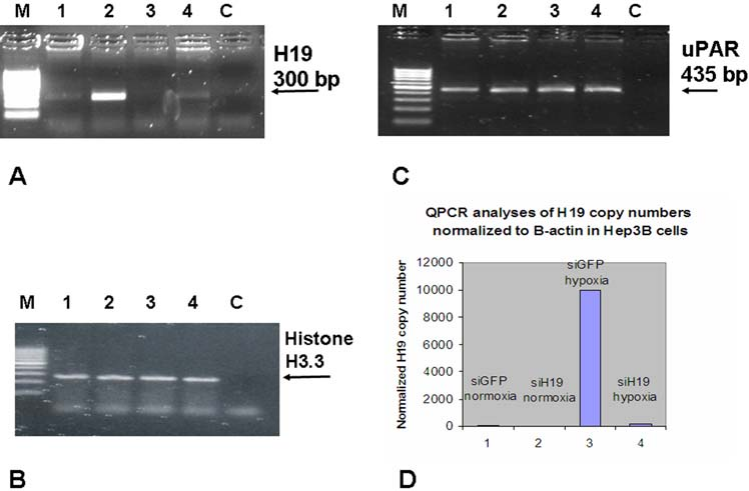
H19 RNA is induced by hypoxic stress in Hep3B cell line and siRNA directed against H19 very efficiently impedes its induction. Hep3B cells were seeded and transfected either with H19 siRNA or luc siRNA . Twenty four hours post transfection, cells were either placed into an aneoropack rectangular jar , or left under normal oxygen concentration. Incubation lasted for 24 hours before RNA extraction. Shown are RT-PCR analyses for H19 RNA (28 PCR cycles). (A): Hep3B transfected with Luc siRNA (lanes 1, 2) and H19 siRNA (lanes 3, 4) both in normal (lanes 1, 3) and hypoxic (lanes 2, 4) culture conditions, respectively. PCR analysis of a house-keeping gene Histone H3.3 (B), and uPAR (C). In (D) QPCR analysis for H19 RNA levels normalized to β-actin in Hep3B cells is depicted.. A Quantitative SYBR Green RT–PCR was performed on human total RNA for both H19 and β-actin to estimate H19 RNA copy number under different manipulations and efficiency of knockdown under hypoxic conditions.

For real hypoxic conditions, only the most effective siRNA was used. Hep3B cells were transfected with H19 siRNA or luc siRNA. Twenty four hours post transfection, cells were either placed into an Aneoropack rectangular jar under hypoxic conditions or continued to grow under normal culture conditions for an additional 24 hours before RNA extraction. Results show a very significant upregulation of H19 RNA in response to hypoxic stress in the cells which were transfected with luc siRNA which served as a control. On the other hand, H19 siRNA nearly completely knocked-down H19 RNA level under normal culture conditions, and totally impeded the upregulation of H19 under hypoxic stress (Fig. 3A). The expression level of two other genes, histone variant H3.3 (Fig. 3B) and urokinase plasminogen activator receptor (uPAR) (Fig. 3C), were not affected by H19 siRNA. These findings suggest that H19 siRNA specifically and effectively knocks-down the level of H19 RNA under both normoxic and hypoxic culture conditions. These effects were also verified through a quantitative RT-PCR on the H19 message levels under normoxic and hypoxic conditions (Fig. 3D).

### Ablation of tumorigenicity by H19 RNA knockdown

**Figure 4:**
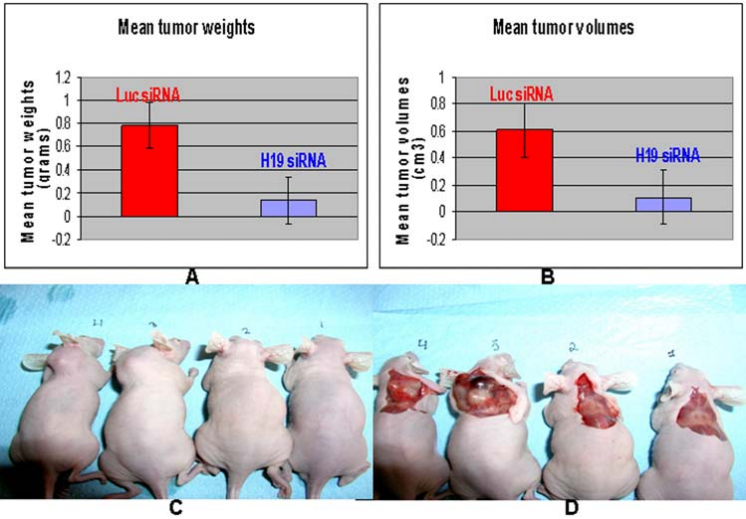
Transient H19 RNA knockdown in Hep3B cells inhibited tumorigenicity in vivo. Hep3B cells were transiently transfected with H19 siRNA3 or Luc siRNA. Forty eight hours post transfection, cells were washed twice with PBS, trypsinized and counted. Equal numbers of cells (1.5×10^6^) were injected subcutaneously into the dorsal part of CD-1 nude mice (n = 7 for both, and 4 for mock transfected). Palpable tumors were observed 15 days post inoculation in mice inoculated with Hep3B, transiently transfected with Luc siRNA. Tumor volumes were followed up and measured using a caliper until day 30 post inoculation, after which mice were sacrificed. Significant (p<0.03) reductions of about 82% of both mean tumor weights (A) (± standard error) and mean tumor volumes (p<0.03) (B) (± standard error) were observed. Values represent end-points just before and after sacrificing animals. Shown are also representative features of tumors in 2 mice of each group (mice 1 and 2 are the H19 siRNA3 treated Hep3B cells, and mice 3 and 4 are the Luc siRNA ) before tumor surgical exposure (C), and after exposure of their internal tumors (D).

Next, we questioned whether H19 RNA is a tumor-associated gene product or whether it is potentially harboring an oncogenic potential by itself. Hep3B cells were transfected in vitro with H19 siRNA3 (Table S1) which proved to be the most potent siRNA in this cell line, or with Luc-siRNA as a control. Forty eight hours post transfection; cells were left to grow in vitro or used for implantation for an ex- vivo study in CD-1 nude mice. For the in vitro assessment, we used the MTS assay, which measures cell proliferation. As shown in (Fig. S4), H19 knock-down did not induce a statistically significant attenuation of cell proliferation of Hep3B cells in vitro. Prior to implantation of the transfected cells in mice, we verify the knocking-down of H19 RNA determined by RT-PCR analysis (data not shown). At this same time point, equal numbers of the ex vivo/in vitro Hep3B transfected and non-transfected cells (1.5×10^6^) were subcutaneously implanted into the dorsa of CD-1 nude mice (groups of 7 mice for each transfected group, and 4 mice for the non-transfected one – similar experiments were repeated two additional times). Results showed that HCC tumors, formed from Hep3B in vitro, transfected with H19 siRNA, encountered a very significant retardation of tumor growth, and in some cases, tumors did not form at all. About a 82% reduction of both mean tumor volumes (Fig. 4B) and mean tumor weights (Fig. 4A) was observed between the two transfected cell lines; however, no significant differences were observed in both mean tumor volumes and weights between tumors formed from untreated Hep3B cells and those that were transfected with Luc siRNA (data not shown). Measurements of two additional experiments showed similar results, when collected two weeks and one month after initial cell innoculation. Furthermore, in one of the experiments, in the four mice receiving Hep3B transfected with the siRNA-3 targeting the H19 message, we did not detect even a trace of tumor as opposed to the five receiving Hep3B transfected with luc siRNA.

### Ectopic H19 expression enhances the tumorigenic potential of bladder carcinoma cells in vivo

**Figure 5:**
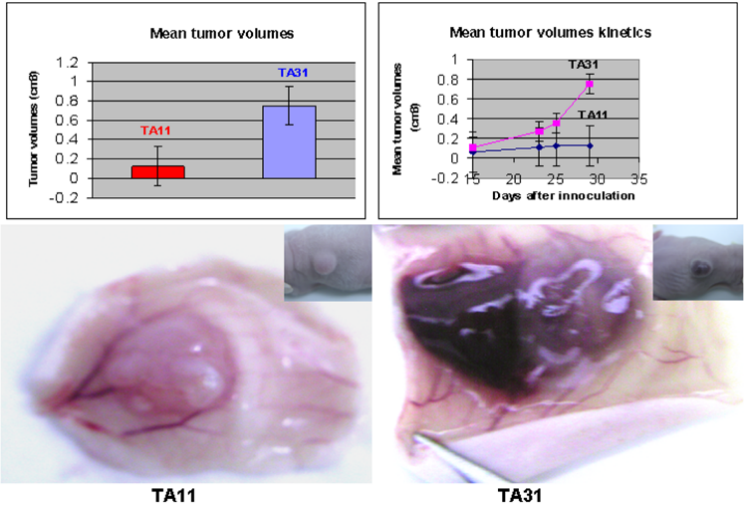
The effect of over-expression of H19 RNA on the tumorigenic potential of bladder carcinoma cells in vivo. Equal amounts (2×10^6^) of TA31^H19high^ and TA11^H19-ve^ cells were implanted subcutaneously to CD-1 mice (n = 5, each). Two weeks later, palpable tumors appeared and tumor volumes were measured for an additional two weeks . Shown are end point measurements of the mean tumor volumes of the two groups (upper left pannel), their mean tumor volumes kinetics (upper right), and a representative gross morphology of tumors derived from the TA11^H19-ve^ (lower left) and TA31^H19high^ cells(down and right).

**Figure 6:**
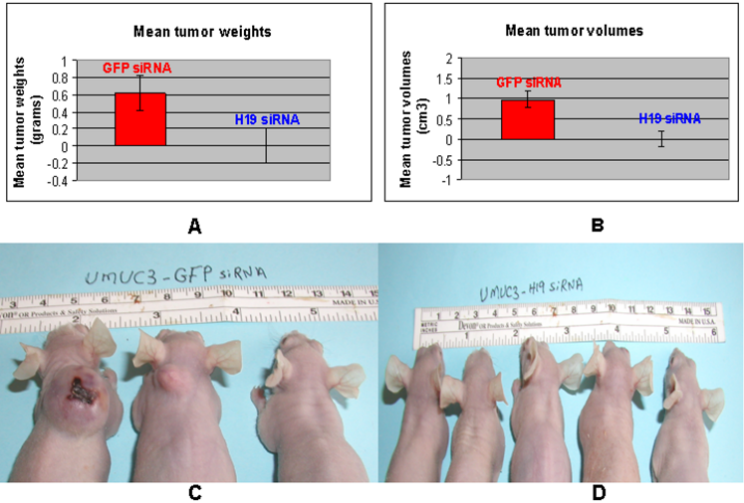
The in vivo effect of H19 silencing on the tumorigenic potential of human bladder carcinoma cells-UMUC3. One million UMUC3 cells were injected subcutaneously to athymic mice (n = 3 for GFP siRNA, and 5 for H19 siRNA ), 48 hours after transiently transfected with siRNAs. Palpable tumors were observed 6 weeks later in 2 out of 3 mice of the GFP siRNA group, while in none of the H19 siRNA group . Mice were sacrificed 8 weeks after inoculation. Mean tumor volumes (B, P<0.05), and mean tumor weights (A, p<0.06) are depicted. Values represent end-points just before and after sacrificing animals. Pictures depict the external features of the tumors in mice inoculated with UMUC3 transfected with GFP siRNA (C), and H19 siRNA (D).

Next, we questioned whether the H19 message affects tumor growth of other cell lineages. Two human bladder carcinoma cells lines, TA11 and TA31, originating from the same parental cell line, T24P, were either negative, (TA11^H19-ve^) or high expressers (TA31^H19high^) of H19, respectively [33]. The T24P cell line was stably transfected with an episomal vector that has an H19 full-length cDNA placed in either the sense direction (the 6-kb H19 fragment spans the complete transcribed region of the human H19 gene and begins 48 nucleotides upstream of the transcriptional start site), creating TA31, or the anti-sense direction (800 bp from 3′ end), creating TA11. These cells were implanted subcutaneously into CD-1 mice. Tumor volumes were measured 15 days post-implantation. As shown in (Fig. 5-top), tumors derived from the TA11^H19-ve^ cells were significantly smaller than those from the TA31^H19high^ cells. Furthermore, the TA31^H19high^ -derived tumors were more vascularized (Fig. 5-bottom). RT-PCR results from the tumors obtained from TA11^H19-ve^ showed that H19 RNA was induced in those tumors as opposed to null expression of H19 RNA in those cells in vitro (data not shown). These results suggest that H19 RNA enhances tumor growth. To directly assess whether H19 expression was also essential for the growth of human bladder carcinoma cells in vivo, we first performed in vitro studies to determine whether siRNA H19 #1 that targets the H19 message, knocks-down H19 RNA in the human bladder carcinoma cell line, UMUC3. As shown, hypoxic conditions increase the H19 RNA level and the siRNA H19 #1 very significantly knocks it down (Fig. S5A). The UMUC3 cells were in-vitro transfected with the siRNA H19 #1 and then implanted in CD-1 mice as was performed with the Hep3B HCC cells in vivo studies (Fig. 6). The knocking-down of H19 message again caused a very significant retardation of tumor growth of the human bladder carcinoma cell line UMUC3 in vivo, as compared to control which was in vitro-transfected with GFP siRNA. The fact that the two different siRNAs targeting the H19 message had an identical effect implies that the phenotype we observed is not related to an off-target effect of the siRNA used.

### Knocking down H19 RNA suppresses p57^kip2^ and abolishes its induction in response to hypoxic stress

**Figure 7:**
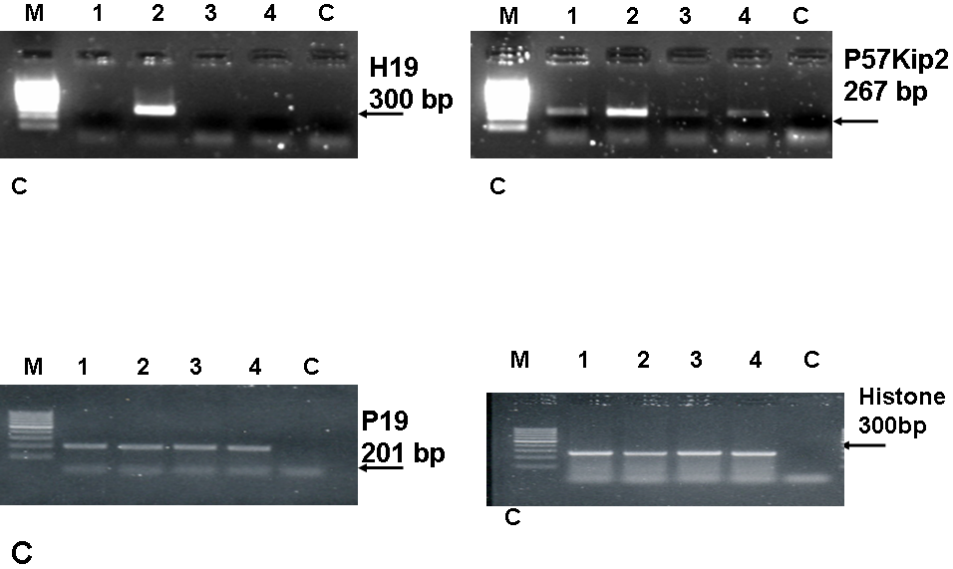
H19 RNA knockdown impedes p57Kip2 mRNA induction in response to hypoxic stress, in Hep3B cell line. Hep3B cells were seeded and transfected either with H19 siRNA or Luc siRNA as described . Shown are RT-PCR analyses for H19 RNA (28 PCR cycles). (A): Hep3B transfected with luc siRNA (lanes 1, 2) and H19 siRNA (lanes 3, 4) both in normal (lanes 1, 3) and hypoxic (lanes 2, 4) culture conditions, respectively. And similar treatments are assessing the mRNA levels of: (B). p19^INK4 ^(C). p57^Kip2^ (D). Histone.

Regional coordination of gene expression and repression is found in many imprinted gene clusters in the mammalian genome and is regulated by imprinting centers or imprinting control regions. A well characterized cluster is one containing the paternally expressed IGF2 and maternally expressed H19 in chromosome 11p15.5. In this study, we chose to test the expression of the cell-cycle inhibitor p57^kip2^; it is the only imprinted cyclin-dependent kinase inhibitor and is located within the same cluster of imprinted genes where H19 is present [39]. Moreover, recent reports have shown a decrease in the expression of p57^kip2^ in HCC compared to normal liver cells [40]. To delineate the effect of H19 levels on p57^kip2^ levels, we knocked-down H19 levels with siRNA H19 #3 in the Hep3B cell line, and siRNA H19 #1 in UMUC3 under both normal and hypoxic conditions. As shown in (Fig. 7B), and (Fig. S5B), the knocking-down of H19 RNA resulted in nearly complete attenuation of p57^kip2^ induction in response to hypoxic stress in both cell lines; moreover, using two different siRNAs targeting H19 strongly indicates the specificity of the results. The expression of p19^INK4^ was not affected as tested in the Hep3B cell line (Fig. 7C).

### H19 knockdown modulates the expression of genes involved in angiogenesis, survival and tumorigenesis in hypoxic stress

To further determine the different targets and pathways which are affected by H19 RNA, we undertook a comprehensive experimental approach by performing a differential gene expression analysis. The effects of the knocking-down of H19 RNA in Hep3B cells have been examined on two gene categories: **1.** Hypoxia-responsive genes modulated by H19 RNA knockdown as shown in Tables 1 and 2; and **2.** Genes not responsive to hypoxia that show modulation of expression by H19 RNA knockdown in hypoxic stress (same Tables-genes marked with a *). We demonstrate that H19 RNA regulates the expression of arrays of gene products that mediate some aspects of the tumorigenic processes that is not only essential for cell survival and angiogenesis, but also accounts for its tumorigenic potential under hypoxic conditions by modulating the mRNA levels of putative oncogenes and tumor suppressor genes. Knocking down H19 RNA reduced the expression of 60 genes and upregulated the expression of 27 genes under these categories.

**Table 1:** Genes reduced by at least two-folds by H19 knockdown in hypoxic stress.

Gene Symbol	Gene name	Folds Change	Functional Category
	**Angiogenesis**		
**TNFAIP1**	Tumor necrosis factor, alpha-induced protein 1 (endothelial)	2.0	Immune response/angiogenesis
**CNN2** *	calponin 2	2.1	Cytoskeleton
**ID2**	inhibitor of DNA binding 2, dominant negative helix-loop-helix protein	2.7	Development
**PRCP**	prolylcarboxypeptidase (angiotensinase C) (not hypoxia)	2.0	Proteolysis
**ANG**	angiogenin	2.5	Angiogenesis
**RNASE4**	ribonuclease, RNase A family, 4	3.0	mRNA cleavage/Angiogenesis
**FGF18**	fibroblast growth factor 18		Growth factor activity
	**Anti-apoptotic/Survival**		
**IER3**	immediate early response 3	2.5	Anti-apoptosis
**PRKCZ**	protein kinase C, zeta	2.2	Anti-apoptotic
**AKT1** *	v-akt murine thymoma viral oncogene homolog 1	3.2	Anti-apoptotic
**MITF** *	microphthalmia-associated transcription factor	2.1	Regulation transcription/differentiation
	**Cytoskeleton**		
**TUBB2A** *	tubulin, beta 2	2.4	Cytoskeleton
**DBN1** *	drebrin 1	3.4	Actin filament organization
**FHOD3** *	formin homology 2 domain containing 3	2.4	Actin cytoskeleton
**FSCN1**	fascin homolog 1, actin-bundling protein (Strongylocentrotus purpuratus)	9.0	Actin cytoskeleton/proliferation
**SNTA1**	syntrophin, alpha 1 (dystrophin-associated protein A1, 59kDa, acidic component)	3.3	Actin cytoskeleton
**MARK4**	MAP/microtubule affinity-regulating kinase 4	3.4	Microtuble bundle formation
**KIF3C** *	kinesin family member 3C	3.0	Microtubule based movement
	**Metabolism**		
**ALDH1A3** *	aldehyde dehydrogenase 1 family, member A3	2.8	Metabolism
**PLA2G4A**	Phospholipase A2, group IV (cytosolic, calcium-dependent)	3.2	Lipid catabolism
**CORO2A**	coronin, actin binding protein, 2A	2.2	Nitrogen compounds metabolism
**PLSCR4** *	phospholipid scramblase 4	2.0	Phospholipids scrambling
**IDH3A**	isocitrate dehydrogenase 3 (NAD+) alpha	3.8	Metabolism
**MPI**	mannose phosphate isomerase	4.4	Carbohydrate metabolism
**AMACR** *	alpha-methylacyl-CoA racemase	2.7	Metabolism
	**Cell cycle/Growth**		
**EBAG9**	Estrogen receptor binding site associated, antigen, 9	5.0	Regulation of cell growth.
**RASSF2**	Ras association (RalGDS/AF-6) domain family 2	2.0	Cell cycle-signal transduction.
**BCL3**	B-cell CLL/lymphoma 3	2.0	Proto-oncogene
**TYMS** *	thymidylate synthetase	2.4	DNA replication
**CCNE2**	cyclin E2	2.3	Cell cycle
	**Signal transduction/transcription**		
**TRAF3IP2**	TRAF3 interacting protein 2	2.6	Signal transducer activity.
**RXRA**	retinoid X receptor, alpha	3.1	Nuclear receptor, transcription activator
**TCF2** *	transcription factor 2, hepatic; LF-B3; variant hepatic nuclear factor	3.3	Transcription factor
**JARID2**	Jumonji, AT rich interactive domain 2	3.8	Transcription/development
**IL1RAP**	Interleukin 1 receptor accessory protein	2.1	Inflamatory response
**UPK1A**	uroplakin 1A	2.1	Signal transduction/deffirentiation
**MYD88** *	Myeloid differentiation primary response gene (88)	2.2	NF-KappaB cascade
**PIP5K2B**	Phosphatidylinositol-4-phosphate 5-kinase, type II, beta	2.0	Signal transduction
**CTNNBIP1**	catenin, beta interacting protein 1	2.3	Wnt receptor signaling pathway
**PEX11A** *	Peroxisomal biogenesis factor 11A	2.3	Signal transduction
**KIAA1196** *	KIAA1196	2.4	Regulation transcription
**ZNF189** *	zinc finger protein 189	2.2	Metal ion binding/ Transcription
	**Miscellaneous**		
**ZNF185**	Zinc fingure protein 185 (LIM domain)	2,3	Metal ion binding
**USP3**	ubiquitin specific peptidase 3	3.4	Ubiquitin dependent protein catabolism
**RAB4A**	RAB4A, member Ras oncogene family	2.2	Protein transport
**ATP13A2**	ATPase type 13A2	2.2	Cation transport/metabolism
**SYNGR3**	synaptogyrin 3	2.6	Membrane protein
**MTMR4**	myotubularin related protein 4	2.2	Phospholipids dephosphorylation
**DAG1** *	dystroglycan 1 (dystrophin-associated glycoprotein 1)	2.1	Laminin receptor activity
**GLTP**	glycolipid transfer protein	2.1	Glycolipid transport
**RAD23B** *	RAD23 homolog B (S. cerevisiae)	2.1	Nucleotide-exision repair
**INPP5A** *	inositol polyphosphate-5-phosphatase, 40kDa	2.4	Hydrolase activity/ Cell comunication
**SNPH** *	syntaphilin	2.5	Synaptic vesicle docking
**FKBP9**	FK506 binding protein 9, 63 kDa	2.8	Protein folding
**TPD52**	'tumor protein D52	3.0	Secretion/deffirentiation
	**Unknown**		
**KIAA0802**	KIAA0802	2.0	
**DENND3**	DENN/MADD domain containing 3	2.4	Unknown
**ZNF668** *	zinc finger protein 668	2.0	Unknown
**KIAA0802**	KIAA0802	3.8	Unknown
**KIAA1598** *	KIAA1598	2.8	Unknown
	Hypothetical protein LOC221362*	2.9	Unknown

* = Hypoxia non-responsive, but shows decreased expression by H19 knockdown under hypoxic conditions

**Table 2:** Genes induced by at least two-folds by H19 knockdown under hypoxic stress.

Gene Symbol	Gene name	Folds Change	Functional Category
	**Putative tumor suppressor activity/ Antiproliferative**		
CYLD*	cylindromatosis (turban tumor syndrome)	3.1	Cell cycle
MTUS1*	mitochondrial tumor suppressor 1	3.2	Receptor activity
PLK2*	polo-like kinase 2 (Drosophila)	2.0	Signal transducer
RGS2	regulator of G-protein signalling 2, 24kDa	2.8	Signal transduction
CAV1*	caveolin 1, caveolae protein, 22kDa	2.4	Cholestrol hemeostasis
BTG3	BTG family member 3	3.6	Antiproliferative
TRIB1	tribbles homolog 1 (Drosophila)	6.9	Regulation MAPK activity
	**Putative Anti-Angiogenesis activity**		
EFNA1	ephrin-A1	3.9	Ephrin receptor binding
ANGPTL4	Angiopoietin-like 4	2.4	Angiogenesis/metabolism
	**Pro-apoptotic factors**		
DDIT3	DNA-damage-inducible transcript 3	4.0	Growth arrest/apoptosis
CASP3	Caspase 3	2.7	Apoptosis
	**Metabolism**		
PYGB*	Phosphorylase, glycogen; brain	2.6	Carbohydrate metabolism
SREBF1	sterol regulatory element binding transcription factor 1	2.6	Metabolism
GPD1L	glycerol-3-phosphate dehydrogenase 1-like	2.0	Metabolism
SMS*	Spermine synthase	2.1	Polyamine metabolism
UAP1	UDP-N-acteylglucosamine pyrophosphorylase 1	4.6	Metabolism
	**Miscellaneous**		
SNAPC1	small nuclear RNA activating complex, polypeptide 1, 43kDa	2.2	Transcription
TSN	Translin	2.0	DNA recombination
RNMT	RNA (guanine-7-) methyltransferase	4.4	mRNA capping
C10orf10	C10orf10	2.5	Fasting induced
MAPK6	mitogen-activated protein kinase 6	3.4	Signal transduction
C1orf9	C1orf9	2.1	Transmembrane protein
NUPL1	Nucleoporin like 1	3.7	Transport
TMEM2	Transmembrane protein 2	4.6	Transmembrane protein
	**Uncharacterized**		
	hypothetical protein DKFZp762E1312*	2.4	Unknown
	hypothetical protein FLJ13611	2.3	Unknown
COL13A1	collagen, type XIII, alpha 1	2.6	Unknown

* = Hypoxia non-responsive, but shows decreased expression by H19 knockdown under hypoxic conditions

Among genes modulated by H19 knockdown, are those that modulate angiogenesis and blood vessels development . H19 RNA knockdown affects the hypoxic responsiveness of the potent angiogenic factors ANG [41], FGF18 [42], prolylcarboxypeptidase (PRCP) or angiotensinase c [43], tumor necrosis factor, alpha-induced protein 1 (endothelial) (TNFAIP1) also known as (B61) [44] Calponin2 (CNN2) [45] and inhibitor of DNA binding 2 (Id2) [46], where they show significantly lower expression levels in response to hypoxia relative to cells possessing H19 RNA. Furthermore, an upregulation of two putative angiogenic inhibitors are noted, namely Angiopoietin-like 4 (ANGPTL4), and Ephrin A1 (EFNA1) [47].

**Figure 8:**
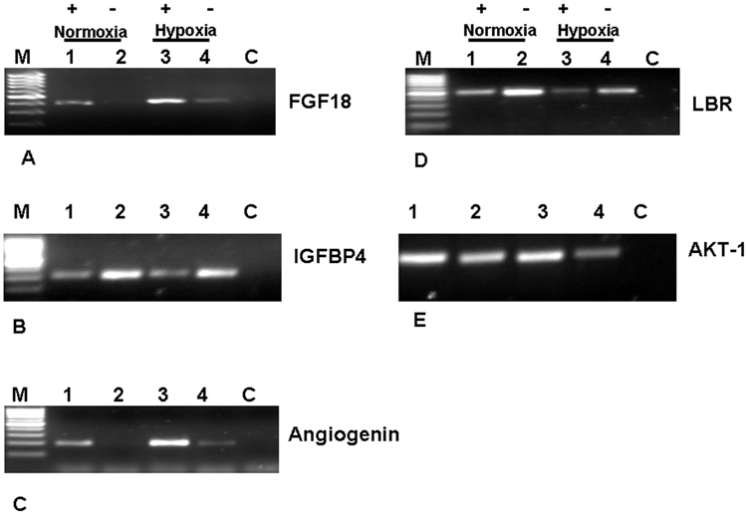
Expression levels of selected genes in Hep3B cells transfected either with H19 and GFP siRNAs under normoxic and hypoxic conditions. A few genes that showed variations as a result of H19 knockdown under different manipulations were selected, based on their potential importance in tumorigenesis, for further RT-PCR analysis to confirm the results of the microarray data. In the figure +denotes (+H19) and –(-H19). The genes chosen were (A)-FGF18; (B)-IGFBP4; (C)-ANG; (D)-LBR; and (E)-AKT1 . The knockdown, of H19 and the RT-PCR integrity are shown in Figure 7.

Moreover, the mRNA levels of many genes implicated in survival/apoptotic decision displayed modulated levels of expression by H19 knockdown in hypoxic stress. H19 knockdown downregulates the mRNA levels of microphthalmia-associated transcription factor (MITF) [48], immediate early response 3 (IER3) [49], protein kinase C, zeta (PRKCZ) [50], B-cell CLL/lymphoma 3 (BCL3) [51], and (Akt-1) [52], all reported as having antiapoptotic function, whereas, the DNA-damage-inducible transcript 3 (DDIT3) also known as (GADD153) is upregulated [53]. Semiquantitative RT-PCR validated changes in the expression levels of some of the genes are shown in (Fig. 8). These included ANG, FGF18 and Akt-1. As shown in (Fig. 8C), knocking down H19 RNA resulted in severe attenuation of ANG induction in response to hypoxic stress, and abolished its expression under normoxic conditions. This also holds true for FGF18 (Fig 8A). Whereas, Akt-1 is not a hypoxia-responsive gene, a pronounced reduction in its level was noted by H19 knockdown in hypoxia (Fig. 8E).

Putative tumor suppressor genes are upregulated by H19 knockdown in hypoxic stress. These include polo-like kinase 2 (PLK2) [54], mitochondrial tumor suppressor 1 (MTUS1) [55], regulator of G-protein signaling 2 (RGS2) [56], cylindromatosis (CYLD) [57], tribbles homolog 1 (TRIB1) [58], caveolin-1 (Cav-1) [59] and BTG family member 3 (BTG3) [60]. On the other hand, genes that are reported to manifest upregulation in a variety of tumors in a number of models and having tumor promoting activities are downregulated. These include the estrogen receptor-binding fragment-associated antigen 9 (EBAG9) [61], fascin homolog 1 (FSCN1) [62], alpha-methylacyl-CoA racemase (AMACR) [63], tumor protein D52 (TPD52) [64], uroplakin 1A (UPK1A) [65], myeloid differentiation primary response gene (88) (MYD88) [66], transcription factor 2 (TCF2) [67], Phospholipase A2, group IV (cytosolic, calcium-dependent) ( PLA2G4A) [68] and those involved in DNA replication and cell cycle progression, thymidylate synthetase (TYMS) and cyclin E2 (CCNE2) [69].

### H19 siRNA very significantly reduced anchorage-independent growth after hypoxia recovery

To test for the functional consequences of the microarray data described above, we then evaluated the effect of H19 suppression on anchorage-independent colony formation in soft agar after hypoxia recovery as an additional assessment of tumorigenicity in vitro. Hep3B cells were exposed to hypoxic stress 4 hours post transfection as indicated in Materials and Methods. Twenty four hours post hypoxic conditions, cells were seeded on soft agar. H19 knocking- down very significantly abrogated anchorage independent growth after hypoxia recovery as both colony number and size were reduced (Fig. S6).

## Discussion

In this study, we used a powerful gene-silencing strategy (RNAi) to knock down the H19 RNA in cellular and animal tumor models. Our results highlight a critical role of H19 RNA in tumor development, and in particular in the growth of HCC. Our results indicate a direct effect of H19 RNA on tumor growth, and a strong association between hypoxia and H19 levels. Altogether, these data coupled with a wealth of information in the literature on the high expression level of the H19 gene in tumor tissue, identify H19 as having a pivotal role in tumor development.

Clonal evolution of tumor cells in the hypoxic microenvironment results from selection of subpopulations that not only resist apoptosis, but also promote the formation of blood vessels. The role of hypoxia in tumorigenesis can be mediated through its effects on oncogene/tumor suppressor genes expression. In this study, we investigate the transcriptional profiles of two populations of Hep3B cells that differ in the RNA level of the hypoxia responsive gene H19, under hypoxic conditions. The effects of loss of H19 gene expression in Hep3B cells have been examined in two gene categories: **1.** Hypoxia responsive genes modulated by H19 knockdown (Tables 1 and 2); and **2.** Genes not responsive to hypoxia, showing modulation of expression by H19 knockdown under hypoxic stress (same Tables-genes marked with an *).

Little is known today about the function of H19 RNA; however, several lines of evidence supporting the involvement of H19 RNA in hypoxic stress response have accumulated during the past few years. We previously identified downstream targets modulated by H19 over-expression in the T24P bladder carcinoma cell line. Functional grouping of genes whose expression were modulated by H19 RNA showed trends towards genes promoting cellular migration, angiogenesis and metastasis. Notably, several of those genes upregulated by the presence of H19 RNA were also known to be induced by hypoxia [33]. Moreover, a proteomic approach has revealed that H19 overexpression in human cancerous mammary epithelial cells positively regulated the thioredoxin gene at the post-transcriptional level, thioredoxin being a key protein of the oxidative stress response and in the reduction of ribonucleotides to deoxyribonucleotides enabling DNA synthesis and the passing of the cells through the S-phase. [70]. Numerous physiological processes involving cellular invasion, blastocyst implantation, and placental development occur under reduced oxygen environments [71]. We have shown that these physiological processes manifested high levels of H19 RNA [23]. Thus, it seemed logical to test whether H19 RNA levels are modulated under hypoxic stress. In one specific tumor such as human hepatocellular carcinoma (HCC), H19 RNA levels are much higher in both the primary and the metastatic tumor than in normal liver (Fig. S1). Our observation that H19 increases under hypoxic stress conditions could imply the relevance of the targets which are those associated with stress activations.

There are several controversial reports in the literature discussing H19 gene function. A tumor suppressor activity was postulated showing that H19 overexpression lowered the tumorigenic properties of cells derived from kidney tumor [72]. Moreover, the H19 gene is frequently inactivated in Wilms tumor [73]. However, H19 is either highly expressed and/or manifest an aberrant allelic pattern of expression in over 30 types of cancer [74], suggesting that H19 may play a role in tumorigenesis.

p57^kip2 ^plays a role in many biological events including differentiation, apoptosis, cell-proliferation and tumorigenesis. Knocking-down H19 RNA resulted in nearly complete attenuation of p57^kip2^ induction in response to hypoxic stress in both Hep3B and UMUC3 cell lines. Although it is not surprising that there may be reciprocity or dependency of expression between imprinting intra-cluster gene expression, the significance of this finding in hepatocarcinogenesis needs further investigation. p57^kip2^, a KIP family cyclin-dependent kinase (Cdk) inhibitor, blocks the cell cycle by acting on multiple cyclin-Cdk complexes. Overall, recent reports on the function of p57^kip2^ point to its role in cell cycle exit rather than a supporter of cellular proliferation. Further investigation is needed to unfold its role in the development of HCC. Furthermore, we also show by RT-PCR analysis that IGFBP4 is upregulated by H19 knockdown irrespective of a hypoxic state (Fig. 8B). IGFBP4 is an inhibitor of the IGF system including IGF2, which reside in close proximity to the H19 gene, and reciprocally imprinted. Although a wealth of information exists on the regulation of those imprinted genes in cis, little is known about trans-regulation. Our observation that knocking down H19 upregulates IGFBP4, which counteracts the tumor promoting activity of IGF2, could provide a link in trans between H19 and IGF2. Moreover, IGFBP4 is shown to have anti-angiogenic and anti-tumorigenic properties [75].

Currently, H19 RNA does not stand alone as an RNA molecule with a direct effect on tumorigenesis. There is evidence that microRNAs can act as oncogenes or tumor suppressors. The miR-17-92 cluster of microRNAs augments the oncogenic effect of the c-Myc in mice [76]. Furthermore, it was found that this cluster, when up-regulated by the expression of c-Myc, down-regulates the expression of the E2F1 protein — one of the transcriptional targets of c-Myc—at the translational level. These findings suggest that the miR-17-92 cluster can act as an oncogene or as a tumor suppressor [76], [77]. Interestingly, the RAS oncogene is regulated by the let-7 microRNA; decreased expression of let-7 microRNA in some human lung tumors causes increased expression of the RAS oncogene and thereby may contribute to tumorigenesis. Altogether, there is a growing amount of evidence supporting the role of RNA molecules as controllers of tumor development. H19 is predicted to have a complex secondary structure which could serve as a substrate for Drosha. A recent report suggested that H19 is a precursor for microRNA 675 [78].

Further studies are necessary to unfold the molecular mechanism(s) controlling the expression of the imprinted gene H19 and its role in tumor development. In this study, we have shown that H19 acts like an oncogene, and we identified some of its downstream targets. Our results, together with recent reports [16], [17] suggest that H19 RNA harbors oncogenic properties, promoting tumorigenesis. Recently, Barsyte-Lovejoy D et al., concluded that the c-Myc oncogene directly induces the H19 RNA and therefore potentiates tumorigenesis. Downregulation of H19 RNA significantly decreased breast and lung cancer cell clonogenicity and anchorage-independent growth [27]. These results together with ours suggest that H19 acts as an oncogene, and we propose that this effect could be triggered by hypoxic stress [79]. Although the oncogenic mechanism(s) of H19 needs further investigation, the fact that H19 is highly expressed in many types of tumors in a relatively high percentage of cases points to H19 as a target for cancer gene therapy.

## Supporting Information

Figure S1 H19 is highly expressed in human HCC. A biopsy from a patient with HCC depicting his primary (A and B) and intra-vascular metastasis (C and D), stained for α-fetoprotein by immunohistochemistry (A and C) or by in-situ hybridization for H19 message (B and D).
(8.70 MB TIF)

Figure S2 RT-PCR analysis for the expression level of H19 in different HCC cell lines: (A) RT-PCR analysis for H19 mRNA was performed on cDNA of the cell lines: Lane 1-SNU 398; Lane-2 SNU 475; Lane-3 Hep3B; Lane -4 HepG2215 (HBV producing cell line); Lane -5 HepG2 (parental to HepG2215) ; Lane -6 FLC4A10 (HBV producing cell line; Lane -7 FLC4 (parental to FLC4A10) ; Lane-8 Huh7; and Lane -9 the bladder carcinoma cell line T24P. C- is the blank. The RT-PCR was carried as described in Materials and Methods. (B) The efficiency of the RT-PCR analysis was tested using GAPDH-specific primers. (C) QPCR analysis of some of the samples mentioned above.
(0.23 MB TIF)

Figure S3 Knocking down kinetics of the H19 gene in Hep3B cell line: H19 and luciferase specific siRNAs were transfected into Hep3B cell line as indicated using the most potent siRNA identified for the H19 gene. At the indicated time points, RNA was extracted and subjected to RT-PCR analysis. Shown are RT-PCR products of H19 (34 PCR cycles) (A), and β-actin (B). Single numbers are RT-PCR products for Hep3B cells transfected with Luc siRNA and odds are for H19 siRNA. C = PCR blank. RNA was extracted at 1, 6, 9 and 12 day's intervals, respectively from transfection.
(1.47 MB TIF)

Figure S4 The effect of knocking-down H19 RNA on the growth of Hep3B cells in vitro: Hep3B cells were seeded and transfected with GFP siRNA or H19 siRNA. Twenty four hours later, cells were washed twice with PBS, trypsinized and counted. 5×103 cells were seeded in quadruples for each group, incubated for 24 hours before MTS assay was performed and measured with an ELISA plate reader. Each bar represents the mean±standard error of 4 replicates.
(0.99 MB TIF)

Figure S5 Knockdown of H19 RNA impedes p57Kip2 induction in response to hypoxic stress in UMUC3 cell line: UMUC3 cells are manipulated as indicated in the legend of Fig1c, with the exception that GFP siRNA is used as a negative control of transfection, and H19 siRNA #1 was used. (A): UMUC3 transfected with GFP siRNA (lanes 1,3) and H19 siRNA (lanes 2,4) both in normal (lanes 1,2) and hypoxic (lanes 3,4) culture conditions respectively, shows again an upregulation of H19 RNA (28 PCR cycles) in response to hypoxic stress (lanes 1,3) and a very efficient knockdown ability (lanes 2,4). (B): RT-PCR analysis of p57Kip2 shows that it is only induced in GFP siRNA negative control treated cells lanes (1, 3), but not in H19 siRNA treated cells (lanes 2, 4). (C): RT-PCR analysis of β-actin.
(1.41 MB TIF)

Figure S6 H19 depletion suppresses anchorage independent colony formation after hypoxia recovery. Equal numbers of Hep3B cells (2.5*103), which were previously transfected with GFP siRNA and H19 siRNA and exposed to hypoxic stress for 24 hours, were seeded into 6-wells plates containing 0.3% top low-melt agarose-0.8% bottom low-melt agarose, 6-wells per each manipulation. After 4 weeks, colony formation was scored microscopically. Each bar represents the mean±standard error of 6 replicates. Significant (p = 0.001) reduction of about 68% of colony forming ability was observed on those transfected with H19 siRNA.
(0.62 MB TIF)

Table S1 siRNA duplexes targeting the human H19 RNA
(0.03 MB DOC)

Table S2 Percent expression of H19 in human tumors
(0.05 MB DOC)

Table S3 Loss of imprinting of the H19 gene in human cancer
(0.05 MB DOC)
