# Supplementary figures and images for: The H19 Non-Coding RNA Is Essential for Human Tumor Growth
Source: PLoS One. 2007 Sep 5;2(9):e845. doi: 10.1371/journal.pone.0000845 (PMC1959184; doi:10.1371/journal.pone.0000845)

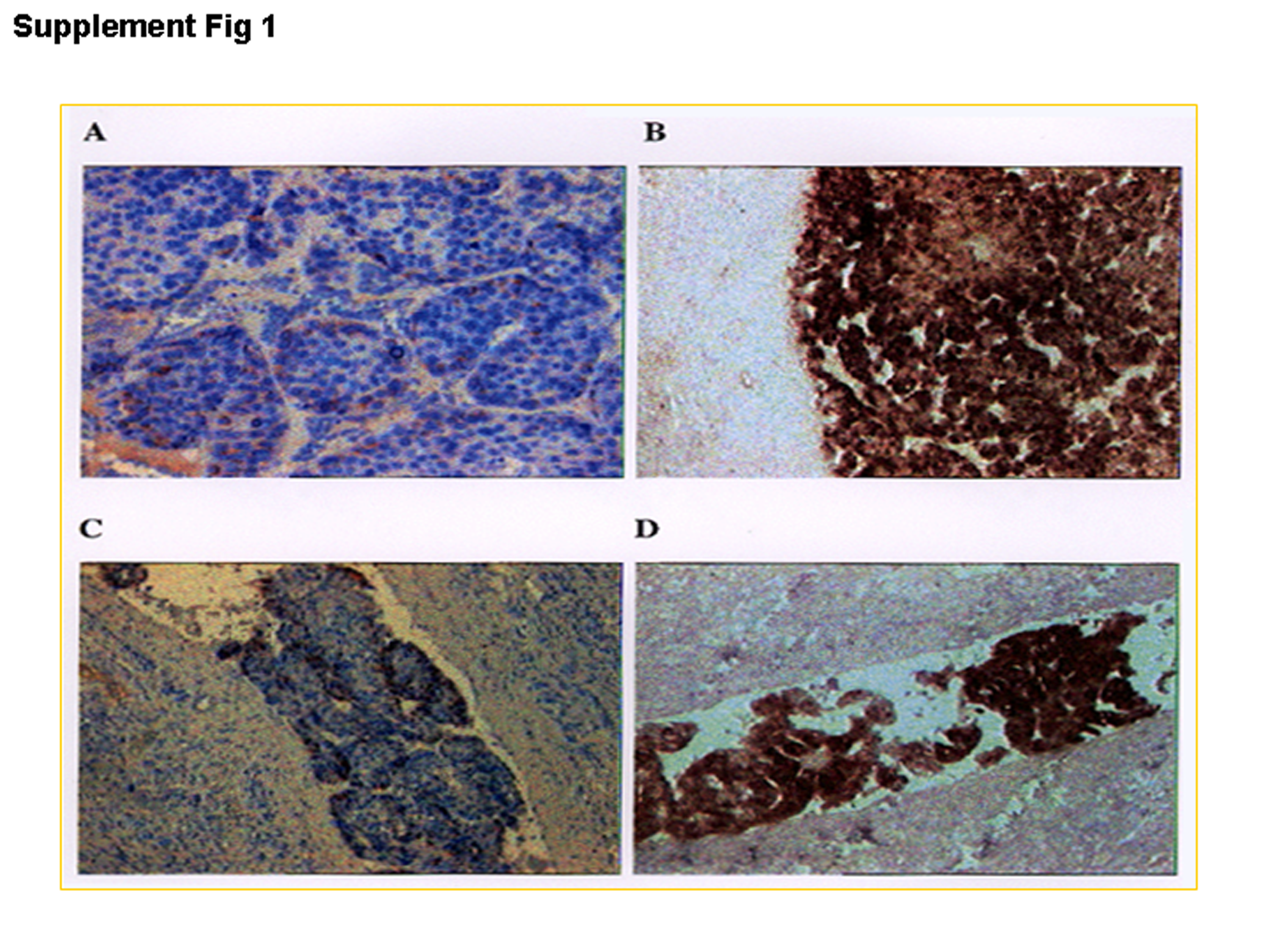

Supplement: Figure S1 — H19 is highly expressed in human HCC. A biopsy from a patient with HCC depicting his primary (A and B) and intra-vascular metastasis (C and D), stained for α-fetoprotein by immunohistochemistry (A and C) or by in-situ hybridization for H19 message (B and D). (8.70 MB TIF) [file pone.0000845.s001.tif]

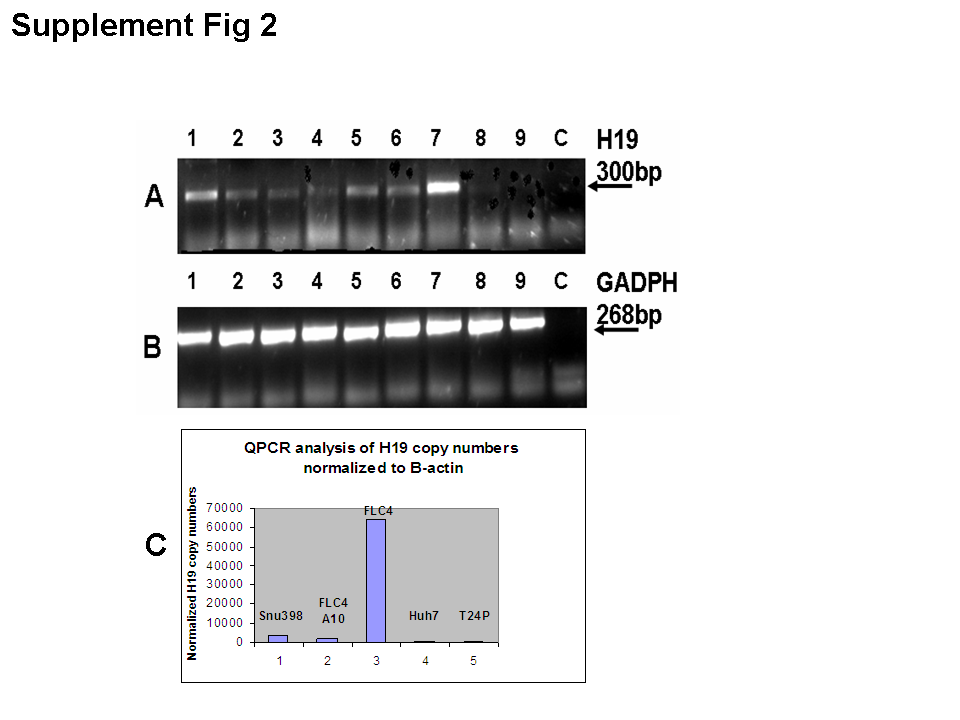

Supplement: Figure S2 — RT-PCR analysis for the expression level of H19 in different HCC cell lines: (A) RT-PCR analysis for H19 mRNA was performed on cDNA of the cell lines: Lane 1-SNU 398; Lane-2 SNU 475; Lane-3 Hep3B; Lane -4 HepG2215 (HBV producing cell line); Lane -5 HepG2 (parental to HepG2215) ; Lane -6 FLC4A10 (HBV producing cell line; Lane -7 FLC4 (parental to FLC4A10) ; Lane-8 Huh7; and Lane -9 the bladder carcinoma cell line T24P. C- is the blank. The RT-PCR was carried as described in Materials and Methods. (B) The efficiency of the RT-PCR analysis was tested using GAPDH-specific primers. (C) QPCR analysis of some of the samples mentioned above. (0.23 MB TIF) [file pone.0000845.s002.tif]

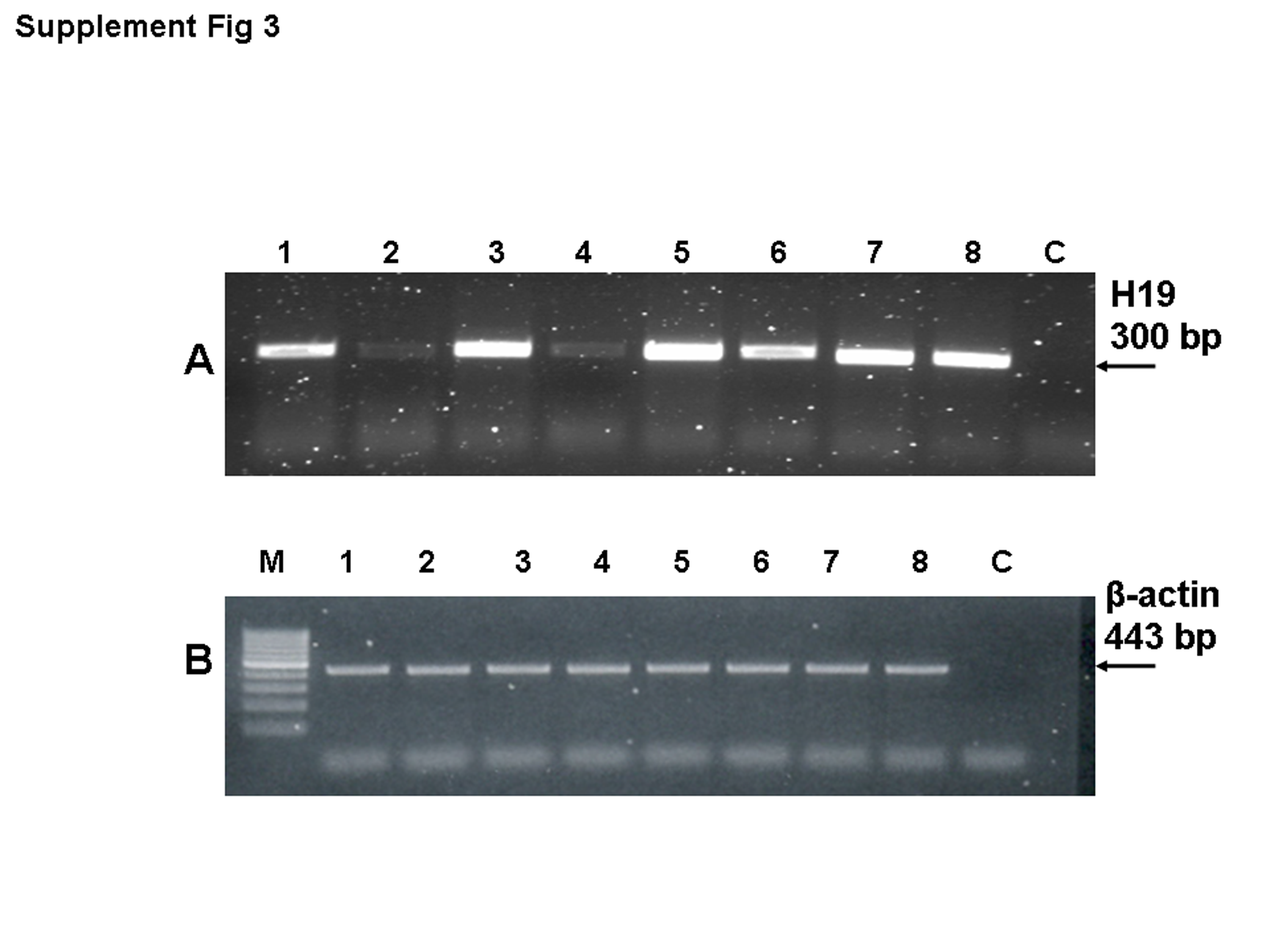

Supplement: Figure S3 — Knocking down kinetics of the H19 gene in Hep3B cell line: H19 and luciferase specific siRNAs were transfected into Hep3B cell line as indicated using the most potent siRNA identified for the H19 gene. At the indicated time points, RNA was extracted and subjected to RT-PCR analysis. Shown are RT-PCR products of H19 (34 PCR cycles) (A), and β-actin (B). Single numbers are RT-PCR products for Hep3B cells transfected with Luc siRNA and odds are for H19 siRNA. C = PCR blank. RNA was extracted at 1, 6, 9 and 12 day's intervals, respectively from transfection. (1.47 MB TIF) [file pone.0000845.s003.tif]

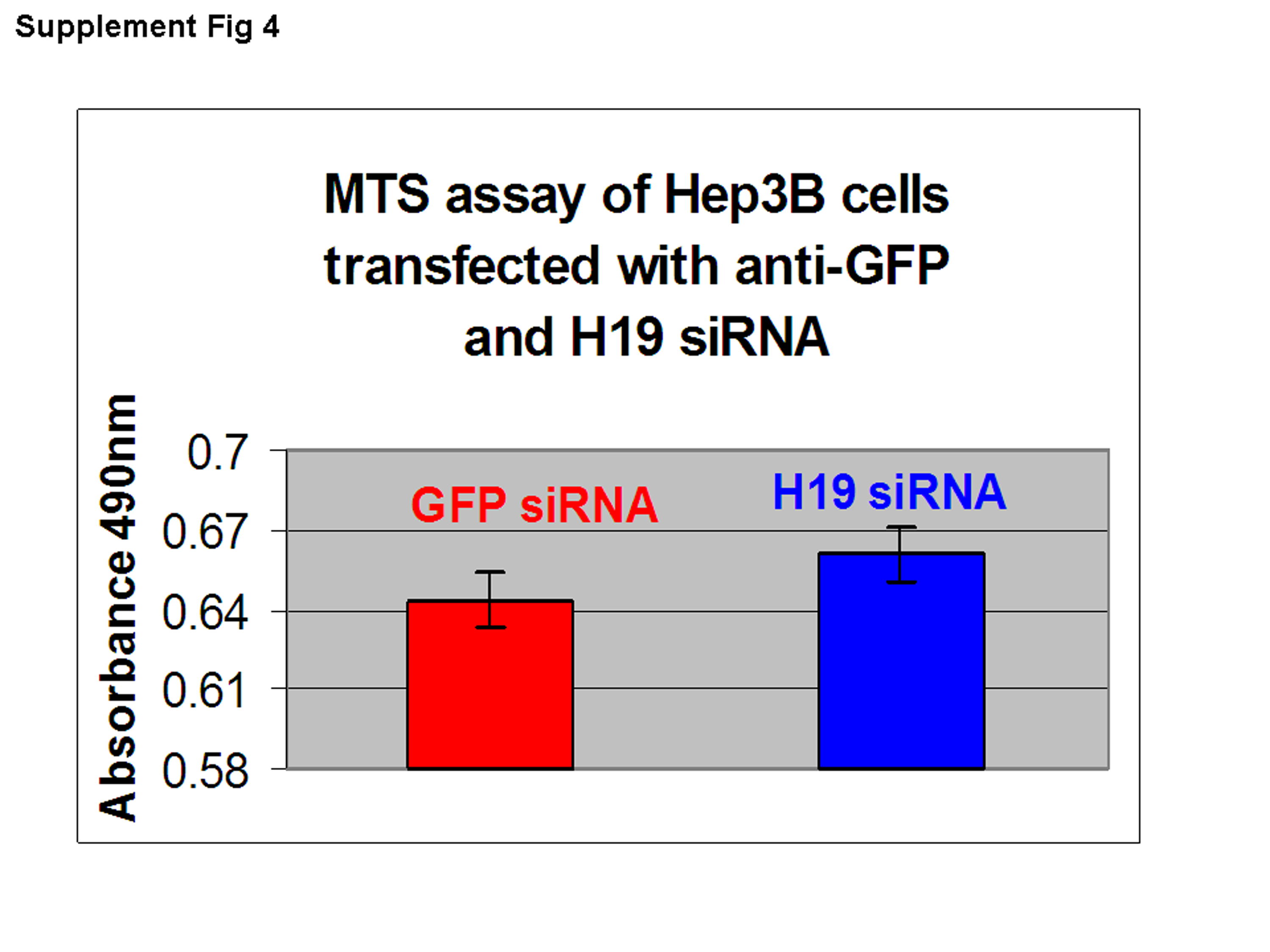

Supplement: Figure S4 — The effect of knocking-down H19 RNA on the growth of Hep3B cells in vitro: Hep3B cells were seeded and transfected with GFP siRNA or H19 siRNA. Twenty four hours later, cells were washed twice with PBS, trypsinized and counted. 5×10^3^ cells were seeded in quadruples for each group, incubated for 24 hours before MTS assay was performed and measured with an ELISA plate reader. Each bar represents the mean±standard error of 4 replicates. (0.99 MB TIF) [file pone.0000845.s004.tif]

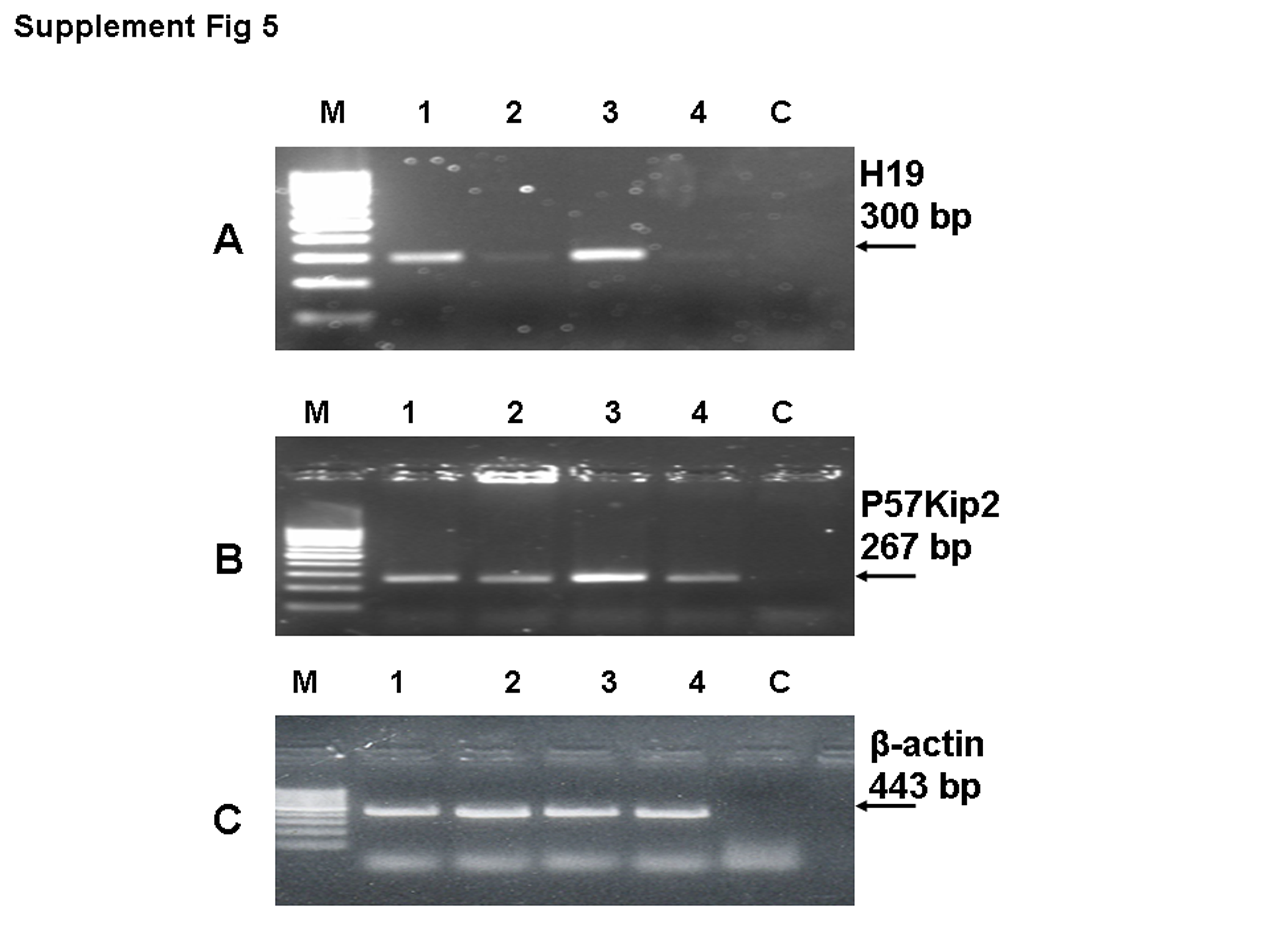

Supplement: Figure S5 — Knockdown of H19 RNA impedes p57Kip2 induction in response to hypoxic stress in UMUC3 cell line: UMUC3 cells are manipulated as indicated in the legend of Fig1c, with the exception that GFP siRNA is used as a negative control of transfection, and H19 siRNA #1 was used. (A): UMUC3 transfected with GFP siRNA (lanes 1,3) and H19 siRNA (lanes 2,4) both in normal (lanes 1,2) and hypoxic (lanes 3,4) culture conditions respectively, shows again an upregulation of H19 RNA (28 PCR cycles) in response to hypoxic stress (lanes 1,3) and a very efficient knockdown ability (lanes 2,4). (B): RT-PCR analysis of p57Kip2 shows that it is only induced in GFP siRNA negative control treated cells lanes (1, 3), but not in H19 siRNA treated cells (lanes 2, 4). (C): RT-PCR analysis of β-actin. (1.41 MB TIF) [file pone.0000845.s005.tif]

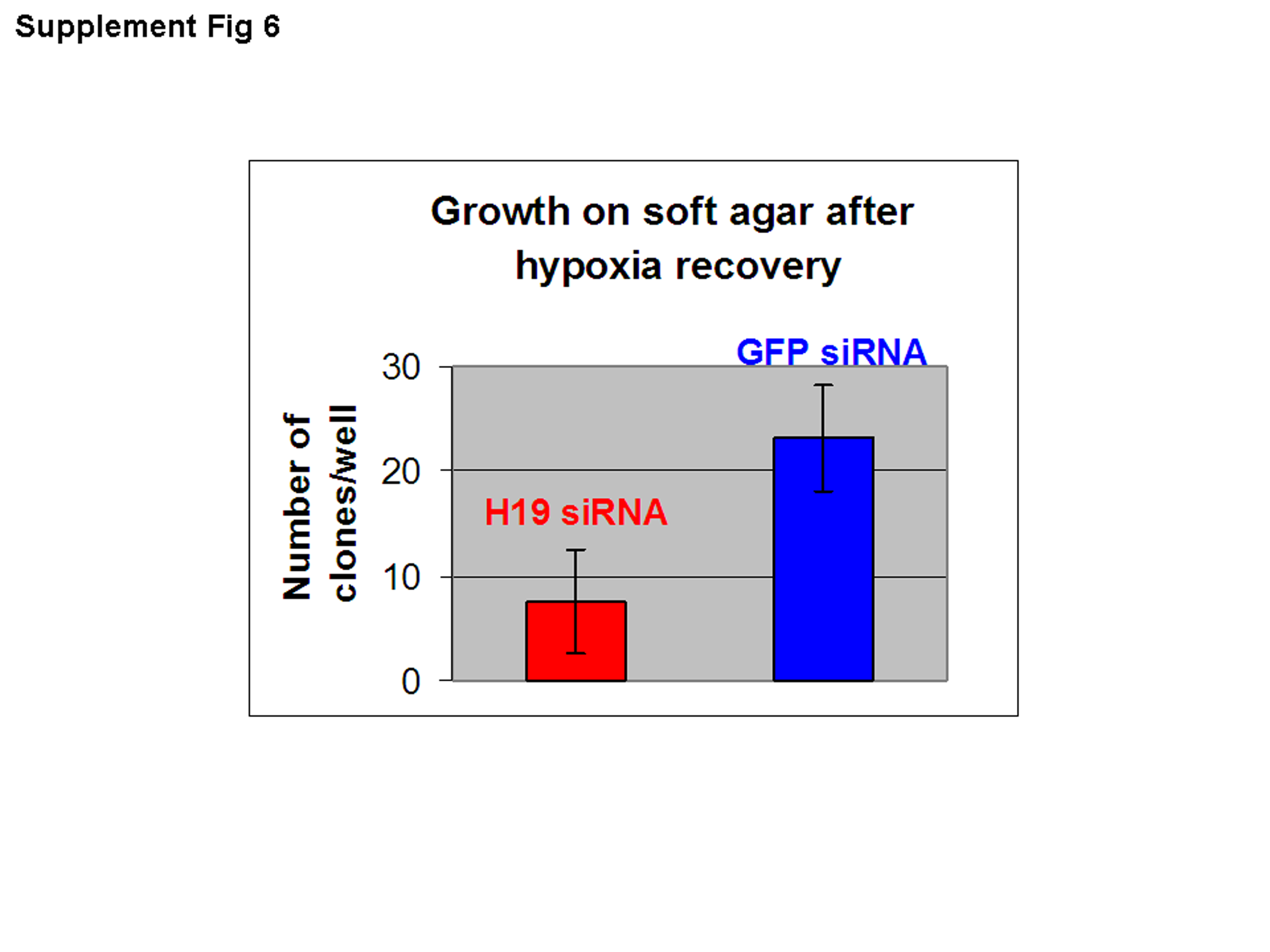

Supplement: Figure S6 — H19 depletion suppresses anchorage independent colony formation after hypoxia recovery. Equal numbers of Hep3B cells (2.5*103), which were previously transfected with GFP siRNA and H19 siRNA and exposed to hypoxic stress for 24 hours, were seeded into 6-wells plates containing 0.3% top low-melt agarose-0.8% bottom low-melt agarose, 6-wells per each manipulation. After 4 weeks, colony formation was scored microscopically. Each bar represents the mean±standard error of 6 replicates. Significant (p = 0.001) reduction of about 68% of colony forming ability was observed on those transfected with H19 siRNA. (0.62 MB TIF) [file pone.0000845.s006.tif]
