# Supplementary material for: The H19 Non-Coding RNA Is Essential for Human Tumor Growth
Source: PLoS One. 2007 Sep 5;2(9):e845. doi: 10.1371/journal.pone.0000845 (PMC1959184; doi:10.1371/journal.pone.0000845)
Supplement: Table S1 — siRNA duplexes targeting the human H19 RNA (0.03 MB DOC) [file pone.0000845.s007.doc]

**Table S1: siRNA duplexes targeting the human H19 RNA**

| **siRNA name** | **Sense sequence** | **Location** |
| --- | --- | --- |
| H19 siRNA1 | 5'-UAAGUCAUUUGCACUGGUUdTdT-3' | Exon 5 |
| H19 siRNA2 | 5'-GCAGGACAUGACAUGGUCCdTdT-3' | Exon 2 |
| H19 siRNA3 | 5'-CCAACAUCAAAGACACCAUdTdT-3' | Exon 5 |
| H19 siRNA4 | 5'-CCAGGCAGAAAGAGCAAGAdTdT-3' | Exon 1 |
| PGL3 siRNA (control) | 5'-CUUACGCUGAGUACUUCGAdTdT-3' | Exon 1 |

Different siRNA duplexes that target H19 RNA were synthesized as ready-to-use duplexes by Proligo (France) with two deoxythymine 3' overhangs in both strands. Unrelated siRNA duplex targets the firefly luciferase gene as a control. Shown are the sequences of the sense strands and their corresponding locations within different exons.
