# Supplementary material for: The H19 Non-Coding RNA Is Essential for Human Tumor Growth
Source: PLoS One. 2007 Sep 5;2(9):e845. doi: 10.1371/journal.pone.0000845 (PMC1959184; doi:10.1371/journal.pone.0000845)
Supplement: Table S2 — Percent expression of H19 in human tumors (0.05 MB DOC) [file pone.0000845.s008.doc]

**Table S2: Percent expression of H19 in human tumors**

| **Percent of H19 over-expression (%)** | **Cancer type** |
| --- | --- |
| 72.5 | Breast adenocarcinoma^1^ |
| 84 | Bladder carcinoma^2^ |
| 67 | Hepatocellular carcinoma^3^ |
| 70 | Uterine tissue myometrium carcinoma^4^ |
| 38 | Lung carcinoma^5^ |
| 75 | Esophageal carcinoma^6^ |
| 65 | Colorectal carcinoma^6^ |
| 75 | Epithelial ovarian cancer^7^ |

**References table 2:**

1. Adriaenssens E, Dumont L, Lottin S, Bolle D, Lepretre A, Delobelle A, Bouali F, Dugimont T, Coll J, Curgy JJ. H19 overexpression in breast adenocarcinoma stromal cells is associated with tumor values and steroid receptor status but independent of p53 and Ki-67 expression. Am J Pathol 1998;153:1597-607.

2. Ariel I, Sughayer M, Fellig Y, Pizov G, Ayesh S, Podeh D, Libdeh BA, Levy C, Birman T, Tykocinski ML, de Groot N, Hochberg A. The imprinted H19 gene is a marker of early recurrence in human bladder carcinoma. Mol Pathol 2000;53:320-3.

3. Ariel I, Miao HQ, Ji XR, Schneider T, Roll D, de Groot N, Hochberg A, Ayesh S. Imprinted H19 oncofetal RNA is a candidate tumour marker for hepatocellular carcinoma. Mol Pathol 1998;51:21-5.

4. Lottin S, Adriaenssens E, Berteaux N, Lepretre A, Vilain MO, Denhez E, Coll J, Dugimont T, Curgy JJ. The human H19 gene is frequently overexpressed in myometrium and stroma during pathological endometrial proliferative events. Eur J Cancer 2005;41:168-77.

5. Kondo M, Suzuki H, Ueda R, Osada H, Takagi K, Takahashi T. Frequent loss of imprinting of the H19 gene is often associated with its overexpression in human lung cancers. Oncogene 1995;10:1193-8.

6. Hibi K, Nakamura H, Hirai A, Fujikake Y, Kasai Y, Akiyama S, Ito K, Takagi H. Loss of H19 imprinting in esophageal cancer. Cancer Res 1996;56:480-2.

7. Tanos V, Prus D, Ayesh S, Weinstein D, Tykocinski ML, De-Groot N, Hochberg A, Ariel I. Expression of the imprinted H19 oncofetal RNA in epithelial ovarian cancer. Eur J Obstet Gynecol Reprod Biol 1999;85:7-11.
