# Supplementary material for: The H19 Non-Coding RNA Is Essential for Human Tumor Growth
Source: PLoS One. 2007 Sep 5;2(9):e845. doi: 10.1371/journal.pone.0000845 (PMC1959184; doi:10.1371/journal.pone.0000845)
Supplement: Table S3 — Loss of imprinting of the H19 gene in human cancer (0.05 MB DOC) [file pone.0000845.s009.doc]

**Table S3:** Loss of imprinting of the H19 gene in human cancer

| **Cancer type** | **Percent Loss of Imprinting (LOI) (%)** |
| --- | --- |
| Head and neck squamous cell carcinoma^8^ | 37.5 |
| Ovarian carcinoma^9^ | 62 |
| Cervical carcinoma^10^ | 34 |
| Osteosarcoma^11^ | 21 |
| Esophageal carcinoma^6^ | 50 |
| Meningioma^12^ | 30 |
| Wilms' tumor^5^ | 29 |
| Lung carcinoma^5^ | 38 |
| Primary choriocarcinoma^9^ | 50 |

**References table3**

8. el-Naggar AK, Lai S, Tucker SA, Clayman GL, Goepfert H, Hong WK, Huff V. Frequent loss of imprinting at the IGF2 and H19 genes in head and neck squamous carcinoma. Oncogene 1999;18:7063-9.

9. Kim HT, Choi BH, Niikawa N, Lee TS, Chang SI. Frequent loss of imprinting of the H19 and IGF-II genes in ovarian tumors. Am J Med Genet 1998;80:391-5.

10. Kim SJ, Park SE, Lee C, Lee SY, Jo JH, Kim JM, Oh YK. Alterations in promoter usage and expression levels of insulin-like growth factor-II and H19 genes in cervical carcinoma exhibiting biallelic expression of IGF-II. Biochim Biophys Acta 2002;1586:307-15.

11. Ulaner A. Loss of imprinting of IGF2 and H19 in osteosarcoma is accompanied by reciprocal methylation changes of a CTCF-binding site. hum mol genet 2003;12:535-49.

12. Muller S, Zirkel D, Westphal M, Zumkeller W. Genomic imprinting of IGF2 and H19 in human meningiomas. Eur J Cancer 2000;36:651-5.
